# Dataset on early growth of cover crops in growth chamber

**DOI:** 10.1016/j.dib.2020.105262

**Published:** 2020-02-08

**Authors:** Gaëlle Damour, Chloé Guérin, Marc Dorel

**Affiliations:** a CIRAD, UPR GECO, F-34398 Montpellier, France; b CIRAD, UPR GECO, F-97130 Capesterre-Belle-Eau, France

**Keywords:** Cover crops, Plant growth, Leaf area, Leaf traits, Plant traits

## Abstract

The data presented in this data paper describe the early growth of cover crop cultivated in growth chamber under non-limiting conditions. Seventeen species of four botanical groups were described after one month of growth. Traits related to plant growth and leaf area development were measured (five traits) and calculated (eight traits). This data set is made available to enable comparisons between dataset, extended analysis and meta-analysis on cover crop traits. The data presented in this article were used on the research article entitled “Leaf area development strategies of cover plants used in banana plantations identified from a set of plant traits’ [1].

**Table:** Specifications Table

Subject	Agronomy, Plant growth and development
Specific subject area	Trait-based description of tropical cover crops growth.
Type of data	Table
How data were acquired	Measurement of plants grown in growth chamber. Leaf areas were assessed using scanner and WinRhizo Pro analytical software (Regent Instruments).
Data format	Raw
Parameters for data collection	Plant were all maintained under non-limiting conditions. The only factor that varies between data is the species identity.
Description of data collection	Seventeen species of cover crops were grown in pots in growth chamber under non-limiting conditions. Ten individuals (=ten pots) per species were used. Plants were harvested just after leaf emergence and after one month of growth; biomasses and leaf surfaces were assessed. Traits related to leaf area development were calculated.
Data source location	City/Town/Region: Experimental station of Neufchateau, Capesterre Belle Eau Country: Guadeloupe, French West Indies Latitude and longitude (and GPS coordinates) for collected samples/data: 16°05′N, 61°35′W
Data accessibility	Repository name: Cirad Dataverse Data identification number:/ Direct URL to data: doi:10.18167/DVN1/MNMXRZ
Related research article	Damour G et al., 2016, Leaf area development strategies of cover plants used in banana plantations identified from a set of plant traits, European Journal of Agronomy, https://doi.org/10.1016/j.eja.2015.12.007

**Table:** 

**Value of the Data** • The data present plant and leaf traits of 17 species of cover crops and could be used by other researchers who need data on these species. • The data enable other researchers to compare their own data with this dataset and to extent their analysis. • These data could be used in meta-analysis on cover crop traits.

## 1 Data description

**Fig. 1:**
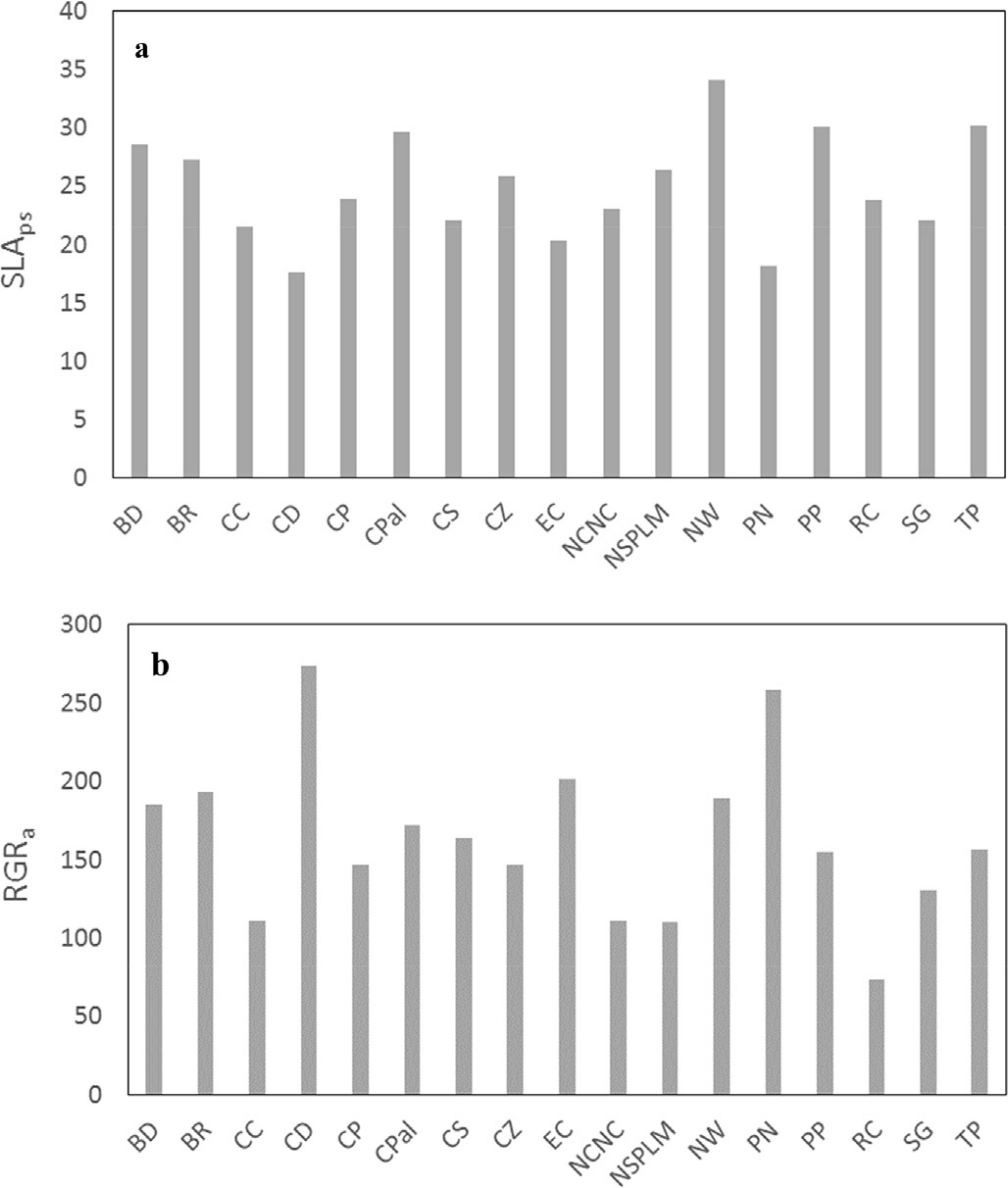
Aboveground specific leaf at the leaf scale (SLA_ps_) (a) and aboveground relative growth rate (RGR_a_) (**b**) for the seventeen species of the dataset.

The dataset presented in this article (doi:10.18167/DVN1/MNMXRZ) provides data on the growth of 17 species of cover crops grown as individuals in growth chamber during one month. It is composed of 17 rows and 18 columns. The first five columns are species names and abbreviation, botanical group and the times of the two plant harvest. The last thirteen columns are aboveground traits: five raw traits (seed mass, leaf area and plant biomass at emergence and after one month) and eight calculated traits (aboveground leaf mass fraction, plant-scale specific leaf area, aboveground leaf area ratio), aboveground absolute and relative growth rates on a biomass basis and on a leaf area basis. Table 1 presents the list of the species names along with their taxonomic groups. Table 2 presents the list of traits along with their units. Fig. 1 represents the diversity of the values obtained for two traits (aboveground specific leaf at the leaf scale and aboveground relative growth rate) for the 17 species of the dataset.

**Table 1:** List of the species available in the dataset.

Abbreviation	Full names	Family	Taxonomic classification
BD	*Bracharia decumbens*	Poaceae	Monocot
BR	*Bracharia ruzziziensis*	Poaceae	Monocot
CC	*Cajanus cajun*	Fabaceae	Dicot
CD	*Cynodon dactylon*	Poaceae	Monocot
CP	*Centrosema pascorum*	Fabaceae	Dicot
CPal	*Crotalaria palida*	Fabaceae	Dicot
CS	*Crotalaria spectabilis*	Fabaceae	Dicot
CZ	*Crotalaria zanzibarica*	Fabaceae	Dicot
EC	*Eleusine coracana*	Poaceae	Monocot
PN	*Paspalum notatum*	Poaceae	Monocot
PP	*Pueraria phaseolides*	Fabaceae	Dicot
NCNC	*Vigna unguiculata* var. CNC	Fabaceae	Dicot
NSPLM	*Vigna unguiculata* var. splm1	Fabaceae	Dicot
NW	*Neonotonia wightii*	Fabaceae	Dicot
RC	*Ricinus communis*	Euphorbiaceae	Dicot
SG	*Stylosanthes guanensis*	Fabaceae	Dicot
TP	*Tagetes patula*	Asteracea	Dicot

**Table 2:** List if the traits provided in the dataset.

Abbreviation	Full trait name	Unit	Nature
SW	seed weight	mg	raw
LA_0_	initial leaf area	cm^2^	raw
LA_1_	total leaf area at one month	cm^2^	raw
BM_a,0_	initial aboveground biomass	g	raw
BM_a,1_	aboveground biomass at one month	g	raw
LMF_a_	aboveground leaf mass fraction	g/g	calculated
SLAF_ps_	plant-scale leaf specific area	m^2^/kg	calculated
LAR_a_	aboveground leaf area ratio	m^2^/kg	calculated
AGR_a_	aboveground absolute growth rate on a biomass basis	mg/j	calculated
AGR_as_	aboveground absolute growth rate on a leaf area basis	cm^2^/j	calculated
RGR_a_	aboveground relative growth rate on a biomass basis	mg/g/j	calculated
RGR_as_	aboveground relative growth rate on a leaf area basis	cm^2^/m^2^/j	calculated
NAR_a_	net assimilation rate	g/m^2^/j	calculated

## 2 Experimental design, materials, and methods

The study was conducted in a growth chamber located at the CIRAD experimental station of Neufchateau in Guadeloupe (French West Indies).

Seventeen cover crop species (Table 2) were grown for one month in pots of 2 L. Each species was grown in ten pots, and one individual per pot was maintained after cotyledons emergence. Details on pot filling materials, seed preparation before sowing and sowing are provided in Damour et al. [1]. The pots were conducted in non-limiting conditions for plant growth during the duration of the experiment: the soil was at field capacity, the air temperature was 24 °C/22 °C (day/night), the light intensity was maintained at 512 μmol photons m^−2^ s^−1^ of photosynthetic active radiation [see 1].

Seed mass (SM) was determined after seed oven-drying at 70 °C until mass stabilization. When the first leaf was fully developed, five replicates of each species were harvested and pooled [see 1, for details on the harvest method]. The **initial leaf area** (LA_0_) was measured with WinRhizo Pro analytical software (Regent Instruments) and the **initial aboveground biomass** (BM_a,0_) was determined after oven-drying of the whole aboveground parts (at 70 °C until weight stabilization). After one month, the five remaining replicates of each species were harvested and pooled. Leaves and stems were separated. The **total leaf area at one month** (LA_1_) was measured with WinRhizo Pro and the leaves and stems biomasses were determined after oven-drying (at 70 °C until weight stabilization). The **aboveground biomass at one month** (BM_a,1_) was calculated as the sum of the leaf and stem biomasses.

Eight functional traits associated to leaf area development were then calculated [1]. The **plant-scale leaf specific area** (SLA_ps_) was calculated as the ratio of the total leaf area and the total leaf mass. The **aboveground leaf mass fraction** (LMF_a_) was calculated as the ratio between the leaf mass and the total aboveground mass. The **aboveground leaf area ratio** (LAR_a_) was calculated as the product of SLA_ps_ and LMF_a_. The **aboveground absolute growth rate** was calculated both on a biomass basis and on a leaf area basis (AGR_a_, AGR_as_ respectively), using the equations:

(1) AGR_a_ = (BM_a,1_ – BM_a,0_)/(t_1_-t_0_)

(2) AGR_as_ = (LA_1_ – LA_0_)/(t_1_-t_0_)

The **aboveground relative growth rate** was calculated both on a biomass basis and on a leaf area basis (RGR_a_, RGR_as_ respectively), using the equations:

(3) RGR_a_ = [ln(BM_a,1_) – ln(BM_a,0_)]/(t_1_-t_0_)

(4) RGR_as_ = [ln(LA_1_) – ln(LA_0_)]/(t_1_-t_0_)

The **net assimilation rate** (NAR_a_) was calculated as the ratio between RGR_a_ and LAR_a_.

All traits, except SLA_ps_ were measured according to the standardized protocols of trait measurements [2].

## Author contributions

**Gaëlle Damour**: Formal analysis, Data curation, Writing, Funding acquisition. **Chloé Guérin**: Methodology, Data acquisition, Formal analysis. **Marc Dorel**: Methodology, Supervision, Funding acquisition.
